# The Effect of Gamification through a Virtual Reality on Preoperative Anxiety in Pediatric Patients Undergoing General Anesthesia: A Prospective, Randomized, and Controlled Trial

**DOI:** 10.3390/jcm7090284

**Published:** 2018-09-17

**Authors:** Jung-Hee Ryu, Jin-Woo Park, Francis Sahngun Nahm, Young-Tae Jeon, Ah-Young Oh, Hak Jong Lee, Jin-Hee Kim, Sung-Hee Han

**Affiliations:** 1 Department of Anaesthesiology and Pain Medicine, Medical Virtual Reality Research Group, Seoul National University College of Medicine, Seoul 03080, Korea; jinaryu74@gmail.com (J.-H.R.); jinul8282@gmail.com (J.-W.P.); hiitsme@hanmail.net (F.S.N.); ytjeon@snubh.org (Y.-T.J.); ohahyoung@hanmail.net (A.-Y.O.); 2 Department of Anaesthesiology and Pain Medicine, Seoul National University Bundang Hospital, Seongnam 13620, Korea; 3 Department of Radiology, Medical Device Research and Development Center, Seoul National University Bundang Hospital, Seongnam 13620, Korea; hakjlee@gmail.com

**Keywords:** preoperative anxiety, virtual reality game, preoperative experience

## Abstract

The use of gamification in healthcare has been gaining popularity. This prospective, randomized, clinical trial was designed to evaluate whether gamification of the preoperative process—via virtual reality (VR) gaming that provides a vivid, immersive and realistic experience—could reduce preoperative anxiety in children. Seventy children scheduled for elective surgery under general anesthesia were randomly divided into either the control or gamification group. Children in the control group received conventional education regarding the preoperative process, whereas those in the gamification group played a 5 min VR game experiencing the preoperative experience. Preoperative anxiety, induction compliance checklist (ICC), and procedural behavior rating scale (PBRS) were measured. Sixty-nine children were included in the final analysis (control group = 35, gamification = 34). Preoperative anxiety (28.3 [23.3–36.7] vs. 46.7 [31.7–51.7]; *p* < 0.001) and intraoperative compliance measured using ICC (*p* = 0.038) were lower in the gamification group than in the control group. However, PBRS (*p* = 0.092) and parent/guardian satisfaction (*p* = 0.268) were comparable between the two groups. VR experience of the preoperative process could reduce preoperative anxiety and improve compliance during anesthetic induction in children undergoing elective surgery and general anesthesia.

## 1. Introduction

Gamification, defined as ‘the use of game design elements in non-game contexts’, has been gaining popularity in healthcare, incorporating elements like points and external rewards to encourage learning [1]. There are many examples of successful implementation of gamification to complement learning in medical education, given the integration of game-like features, including competition, narrative, leaderboards, graphics, and other game design elements, that may induce greater motivation and engagement [2,3]. In addition to medical education, gamification has been reported to be effective in patient education with various clinical conditions, such as psychological therapy and physical therapy [4]. 

With the recent advancement in technology, highly immersive, vivid virtual reality (VR) systems have been introduced into patient education [5,6]. The main characteristics offered by VR technology is the high immersion and real-time interaction [7]. A sense of presence in the VR is considered the major mechanism that enables anxiety to be felt. Meta-analysis demonstrated a positive relationship between the sense of presence and anxiety during VR exposure therapy to manage anxiety disorders [8]. Another study also examined the influence of real or virtual social stimuli on stress reactivity with a 5 min public speaking task, and the result showed comparable increases in salivary cortisol and cardiovascular reactivity in both the real and the virtual public speaking group [9]. These results show a promising future for the application of VR in various therapies. In a previous investigation, a 360 degree VR tour of the operating room was shown to be useful in providing a consistent, vivid, and immersive experience of the preoperative process, significantly reducing preoperative anxiety without physical and financial limitations [6]. 

Distress during the preoperative period usually leads to preoperative anxiety, with incidences of 40–60% [10]. Preoperative is also associated with adverse consequences, such as emergence delirium, higher postoperative pain, and postoperative maladaptive behavioral changes [11,12,13]. The authors hypothesized that the integration effect of gamification (motivation and engagement) and VR technology (immersion and reality) may provide a novel platform to increase active participation and motivation in pediatric patients. Therefore, this prospective, randomized, controlled study was performed to identify whether gamification of the preoperative process—via VR gaming—would induce a reduction in preoperative anxiety and an improvement in the compliance of pediatric patients undergoing general anesthesia and elective surgery.

## 2. Methods

### 2.1. Study

The protocol of this prospective, randomized, and controlled trial was approved by the institutional review board (IRB) of Seoul National University Bundang Hospital (IRB number: B-1801-445-302) and registered at University hospital Medical Information Network (UMIN) Clinical Trials Registry (registration number: UMIN000031252). Written informed consent was obtained from all parents/guardians of pediatric patients, and children aged 7 years or older signed additional agreements directly after receiving detailed instructions with their parents/guardians. This study was conducted at the Seoul National University Bundang Hospital between February and April of 2018. 

### 2.2. Patients

A total of 70 American Society of Anesthesiologist (ASA) physical status I or II children, aged 4–10 years, undergoing elective day surgery and general anesthesia were enrolled in this study. The exclusion criteria were as follows: children requiring major surgery or postoperative intensive care; those with history of prematurity or congenital disease; those with hearing impairment; those with cognitive deficits or cognitive and intellectual developmental disabilities; those with prior experience of anesthesia; those taking psychoactive medications; and those with a history of epilepsy or seizure. Using a computer-generated randomization code (Random Allocation Software version 1.0; University of Medical Sciences, Isfahan, Iran), the enrolled patients were randomly allocated to one of two groups—the control or gamification group. Randomization was performed by an independent anesthesiologist who was only responsible for patient assignment 2 h before the arrival of patients in the reception area of the operating room.

### 2.3. VR Game

**Figure 1:**
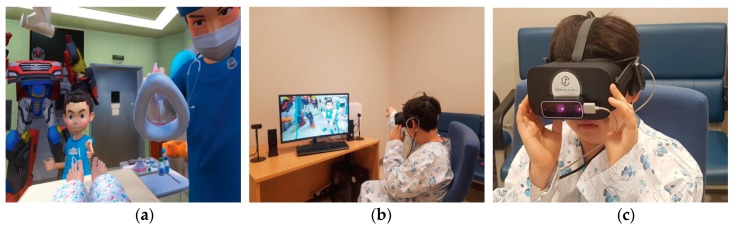
Virtual reality video game. (**a**) Players experience general anesthesia induction in a 360 degree, three-dimensional virtual environment, through first-person viewpoint; (**b**) can interact with virtual devices; (**c**) with a head-mounted VR display and a hand and finger motion controller.

A 5 min VR game was produced in collaboration with a VR game producing company (JSC GAMES, Seoul, Korea). In the VR game, pediatric patients experienced the preoperative process and general anesthesia induction in a 360 degree, three-dimensional virtual environment, through first-person perspective (Figure 1a). Game elements, including virtual world, progression, exploration, challenge, and rewards, were incorporated. The player is first asked to change into a surgical gown after the confirmation of patient ID. Upon confirmation, the player is transported to the operating room (OR). The player is given the opportunity to explore and interact with the OR environment, including monitoring devices—i.e., ECG, saturation monitor, and non-invasive blood pressure (Figure 1b). When the player selects a device, a detailed description of its function is presented. In addition, the player is given the opportunity to learn how to properly breathe with the facial oxygen mask. The player is given the opportunity to choose a mask, based on the preferred fragrance, which included orange, cherry, or bubble gum. Following the instructions prompted in the game, the player is faced with challenges to defeat the germ monster. Each time the player advances to the following preoperative step, they are rewarded ‘health points’. During the game, Chatan and Ace, famous animation characters of an animated film ‘Hello Carbot’ (ChoiRock Contents Factory, Seoul, Korea), explains the process in detail, encouraging players to cooperate appropriately. Permission to use these animation characters had been obtained (licensing agreement with ChoiRock Contents Factory). A head-mounted VR display, Oculus rift (Oculus VR, Menlo Park, CA, USA), and a hand and finger motion controller, Leap Motion Controller (Leap Motion, San Francisco, CA, USA) were used to play the VR game (Figure 1c). 

### 2.4. Intervention

Baseline modified Yale Preoperative Anxiety Scale (m-YPAS) was performed by a blinded single evaluator at the time of admission. After measurement of the baseline m-YPAS, randomization was performed by an independent anesthesiologist, not otherwise involved in the trial using a computer-generated randomization code (Random Allocation Software version 1.0; University of Medical Sciences, Isfahan, Iran) with an allocation ratio of 1:1. An opaque envelope containing sequential numbers was transferred to a researcher, and the intervention was performed in an empty room 1 h prior to entering the operating room. For pediatric patients and parents/guardians in the control group, the conventional mode of education about the preoperative process was provided. Children in the gamification group played a 5 min VR game to experience the preoperative process. After the intervention, children and parents/guardians were encouraged to ask questions about the preoperative procedure and anesthesia to the researcher.

### 2.5. Anesthesia

Without premedication, children were transported to the operating room with their parent/guardian. Monitoring devices, including ECG, non-invasive blood pressure, and oxygen saturation, were applied. Induction of general anesthesia was performed by an anesthesiologist with at least 4 years of experience using intravenous (thiopental sodium, 5 mg/kg) or inhalation agent (sevoflurane) with a facial mask in oxygen. After confirming the loss of consciousness and disappearance of eyelid reflex, the concentration of sevoflurane was increased and intravenous muscle relaxant (rocuronium, 0.3–0.6 mg/kg) was administered. Then, parent/guardian was guided to the waiting room. Endotracheal intubation was performed following sufficient muscle relaxation. During the operation, anesthetic depth was maintained with 1–1.5 MAC sevoflurane. Reversal agents of muscle relaxant (glycopyrrolate and neostigmine) were administered after the surgery. Extubation was done, and patients were transferred to the post-anesthesia care unit (PACU).

### 2.6. Study Outcomes

All outcomes were assessed by a blinded single evaluator to exclude any possible interrater bias. The primary outcome was preoperative anxiety, which was measured using the validated Korean version of the modified Yale Preoperative Anxiety Scale (m-YPAS; Supplementary Table S1) [14]. The m-YPAS was evaluated twice: once at the time of admission prior to the intervention (baseline) and once just before the transportation from the reception area to the operating room for anesthetic induction (pre-anesthetic). Secondary outcomes included induction compliance checklist (ICC; Supplementary Table S2) [15], procedural behavior rating scale (PBRS; Supplementary Table S3) [16], and parent/guardian’s satisfaction scores. ICC represents compliance of patients during induction of anesthesia, and PBRS describes the behavior in a stressful medical procedure. High scores of m-YPAS, ICC, and PBRS represented high anxiety, poor compliance, and increased stress, respectively. Parents/guardians were guided to the waiting room right after the induction of anesthesia, and they were asked to rate the satisfaction about the overall preoperative process of general anesthesia using a numerical rating scale (101 NRS; 0, very dissatisfied; 100, very satisfied).

### 2.7. Statistical Analysis

Power analysis was performed using G*Power 3.1.2 (Heinrich-Heine University, Düsseldorf, Germany). A previous study reported that the mean (SD) of m-YPAS score was 30.1 (8.4) in the control group undergoing elective surgery [17]. For clinical significance of the effect of the VR game, a reduction of 20% of the m-YPAS score was necessary. A sample size of 35 children per group was calculated with power of 0.8, significance level of 0.05, and 10% dropout rate. SPSS version 21.0 (SPSS Inc., IBM, Chicago, IL, USA) was utilized for all statistical analyses. The test of normal distribution was assessed using Shapiro-Wilk test. Continuous data (age, height, weight, BMI, induction time, anesthesia time, operation time, m-YPAS, PBRS score, and satisfaction scores) are presented as the median (interquartile range [IQR]), and categorical variables (gender, ASA physical class, induction agent, type of surgery, ICC score) are shown as numbers (%). Mann–Whitney U test was used for the analysis of continuous variables, and χ^2^ test or Fisher’s exact test was used for categorical variables. A full analysis set was used for data analysis. All of the reported *p*-values are two-sided. A *p* value of less than 0.050 was considered to indicate statistical significance. 

## 3. Results

**Figure 2:**
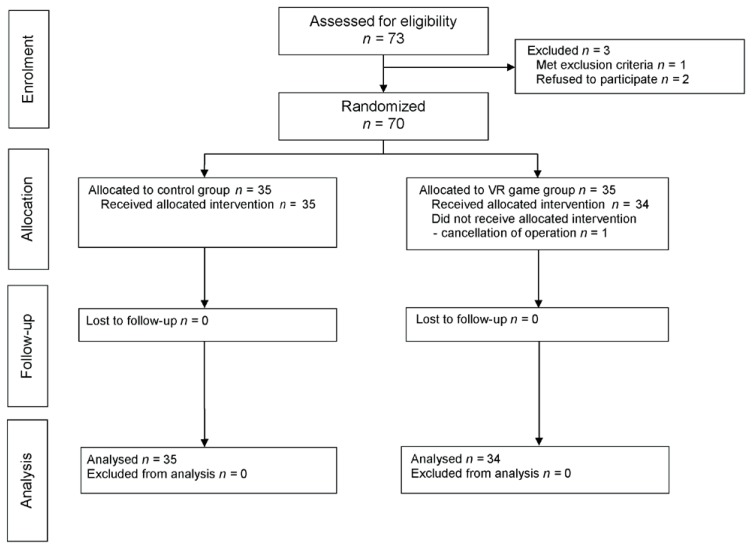
Consort diagram.

Of the 73 screened pediatric patients, 3 were excluded (2 declined to participate, and 1 met the exclusion criteria for cognitive deficit); the remaining 70 patients were randomly allocated to one of two groups: the control or gamification group (35 patients in each group). One child in the gamification group was excluded after randomization because the operation was cancelled. Thus, 69 children completed the study (Figure 2). There were no significant differences in the characteristics of patients, anesthesia, and surgery between the two groups (Table 1).

There was no substantial difference between the two groups with respect to the baseline anxiety before the intervention. However, the pre-anesthetic m-YPAS scores of the gamification group were significantly lower than those of the control group after the intervention (28.3 [23.3–36.7] vs. 46.7 [31.7–51.7]; *p* < 0.001, Table 2). Moreover, the changes of m-YPAS scores before and after the intervention were also significantly different between the two groups (−22.5 [−29.6–14.2] vs. 0 [−20–4.2]; *p* = 0.002, Table 2). 

Compliance during induction of anesthesia was measured with ICC; a greater number of pediatric patients in the gamification group showed better compliance than in the control group (*p* = 0.038, Table 2). However, stressful behaviors during anesthetic induction that were evaluated with PBRS were comparable between the two groups (*p* = 0.092, Table 2). Parent/guardian satisfaction about the overall preoperative process of general anesthesia was similar in both groups (*p* = 0.268, Table 2). This section may be divided by subheadings. It should provide a concise and precise description of the experimental results, their interpretation as well as the experimental conclusions that can be drawn.

## 4. Discussion

To the best of our knowledge, this is the first clinical study showing the outcome of using gamification—via VR technology—to educate pediatric patients in preparation for general anesthesia. The result of the current study showed that VR game reduced preoperative anxiety and improved compliance during anesthetic induction in children undergoing elective surgery and general anesthesia. 

Gamification—as a means to educate patients to improve health outcomes—has previously been introduced and investigated, especially in the field of psychological and physical therapy [4]. Preoperative anxiety is a major concern in pediatric anesthesia because it may induce severe distress and various negative effects in children [10]. With the advancement of VR technology, a recent trial incorporated VR technology and found that preoperative anxiety was reduced by 30% in the VR group compared with the conventional education group [6]. During cognitive behavioral therapy for anxiety disorder, patients were exposed to anxiety-provoking situations, both in real life or via VR experience. They showed that the sense of presence is positively correlated with the level of anxiety [8]. Additionally, a comparable increases in stress response, such as salivary cortisol and cardiovascular reactivity, were observed in both the real and VR groups, suggesting the usefulness of VR applications in anxiety therapy [9]. Therefore, VR exposure can be considered as a practical alternative to traditional exposure, since it offers more control over the anxiety level of the stimulus. The current study evaluated the effects of gamification—via VR gaming—on preoperative anxiety. The result showed that experiencing the preoperative process via VR game may effectively reduce anxiety in pediatric patients (about 40%). The result of the current study may be explained by the combined effect of gamification (motivation and engagement) and VR experience (immersion and reality).

High ICC scores indicate less behavioral compliance [15], and the ICC scores of the present study were stratified into three categories (perfect, moderate, and poor) for the purpose of analysis. Children with VR game experience were more compliant during the induction of anesthesia than those with conventional education. Pediatric patients who played the VR game may be more familiar with the operating room environment and the preoperative process than those who received the conventional education, leading to higher compliance during the induction of anesthesia.

The PBRS score was measured during the induction of anesthesia to assess the degree of distress in children. However, there was no difference in PBRS between the two groups. This phenomenon may be because PBRS was more useful for assessing the distress and pain during painful procedures and postoperative period [16]. 

This study has some limitations. First, this study used the conventional preoperative education method in the control group. This is because our institution had been using this method to mitigate preoperative anxiety in children. The use of the same interactive VR intervention without the gamification component in the control group could reveal the true effect of gamification on preoperative anxiety. Second, there was no data available regarding the use of VR intervention or users’ experiences, such as a measurement of the sense of presence. The current study was performed not to measure the effectiveness of VR intervention, but to identify whether gamification of the preoperative process, using VR, would reduce preoperative anxiety. Additionally, the sense of presence is measured with presence questionnaires [18], which is not clinically suitable for children undergoing anesthesia and surgery. Third, repeated measures analysis of variance (RM ANOVA) is the standard method for randomized controlled trials; however, it was not used for the analysis of the data in this study since the residual of the data did not follow a normal distribution. Therefore, the changes in preoperative anxiety (before and after treatment) were analyzed, and we found significant differences between the groups. Fourth, there is the possibility that the baseline m-YPAS may have been affected by randomization since children may figure out or explicitly ask the staff about their allocation at the time of collecting the pre-measurements. However, the baseline m-YPAS was collected before randomization, and the assessor was blinded to patient allocation.

## 5. Conclusions

Gamification—via the use of VR game—on the experience of the perioperative process appears to reduce preoperative anxiety and improve compliance during anesthetic induction in children undergoing elective surgery and general anesthesia. However, further study with the same interactive VR intervention without the gamification component as the control group may be necessary to investigate the true effect of gamification on preoperative anxiety. Gamification of the preoperative process may be a necessary component of pediatric patient education in reducing preoperative anxiety. It is a low-cost and easy-to-use tool with potential benefits. Nonetheless, a further investigation is needed to measure the effectiveness of users’ experiences with gamification. 

## Figures and Tables

**Table 1:** Patients, anesthesia and surgery characteristics.

	Control Group (*n* = 35)	Gamification Group (*n* = 34)	*p* Value
Age (year)	6 (5−8)	5 (5−7)	0.170
Height (cm)	116.7 (109.8–127.3)	114.7 (108.7–125.7)	0.618
Weight (kg)	21.3 (18.9–28.6)	20.9 (18.4–28.9)	0.529
BMI (kg/m^2^)	16.8 (15.7–17.6)	16.3 (15.2–17.3)	0.400
Gender (M/F)	22 (63)/13 (37)	18 (53)/16 (47)	0.469
ASA physical class (I/II)	34 (97)/1 (3)	33 (97)/1 (3)	>0.999
Induction time (min)	6 (4.5–8)	6 (5–7.8)	0.789
Anesthesia time (min)	40 (35–50)	42.5 (35–50)	0.479
Operation time (min)	20 (17.5–25)	20 (15–30)	0.997
Type of surgery			0.569
Otolaryngeal	12 (34)	17 (50)	
Ophthalmic	14 (40)	12 (35)	
Dental	4 (11)	2 (6)	
Others	5 (15)	3 (9)	

VR: virtual reality; ASA: American Society of Anesthesiologist; Induction time: time from the entrance into the operating room to intubation. Data are expressed as median (interquatile range [IQR]) or numbers (%).

**Table 2:** Preoperative anxiety, induction compliance, and stressful behaviors of children and parental satisfaction.

		Control Group (*n* = 35)	Gamification Group (*n* = 34)	*p* Value
m-YPAS	baseline	50.0 (43.3–65)	51.7 (46.7−67.5)	0.389
	preanesthetic	46.7 (31.7–51.7)	28.3 (23.3−36.7)	<0.001
	difference	0 (−20–4.2)	−22.5 (−29.6–−14.2)	0.002
ICC score	perfect (0)	19 (54)	27 (79)	0.038
	moderate (1–3)	13 (37)	7 (21)	
	poor (>4)	3 (9)	0 (0)	
PBRS score		1 (0–2)	0 (0–1)	0.092
Satisfaction Score (101 NRS)		100 (90–100)	100 (90–100)	0.268

m-YPAS: modified Yale Preoperative Anxiety; ICC: induction compliance checklist; PBRS: procedural behavior rating scale; NRS: numerical rating scale (0, very dissatisfied; 100, very satisfied). Data are expressed as median (IQR) or numbers (%).

## Supplementary Materials

The following are available online at http://www.mdpi.com/2077-0383/7/9/284/s1, Supplementary Table S1: modified Yale Preoperative Anxiety Scale, Supplementary Table S2: induction compliance checklist, Supplementary Table S3: procedural behavior rating scale.
