# Supplementary material for: The Effect of Gamification through a Virtual Reality on Preoperative Anxiety in Pediatric Patients Undergoing General Anesthesia: A Prospective, Randomized, and Controlled Trial
Source: J Clin Med. 2018 Sep 17;7(9):284. doi: 10.3390/jcm7090284 (PMC6162739; doi:10.3390/jcm7090284)
Supplement: Supplementary file 1 [file jcm-07-00284-s001.zip › Supplementary Table S1.doc]

**mYPAS (modified Yale preoperative anxiety scale)**

**A. Activity _____**

**B. Vocalizations _____**

**C. Emotional Expressivity _____**

**D. State of Arousal _____**

**E. Use of Parent _____**

**A. Activity**

0. Can’t code (child not visible)

1. Looking around, curious, playing with toys, reading (or other age appropriate behavior); moves around holding area/ treatment room to get toys or go to parent; may move toward OR equipment

2. Not exploring or playing, may look down, may fidget with hands or suck thumb (blanket); may sit close to parent while wait­ing, or play has a definite manic quality

3. Moving from toy to parent in unfocused manner, nonactivity derived movements; frenetic/frenzied movement or play; squirming, moving on table, may push mask away or clinging to parent

4. Actively trying to get away, pushes with feet and arms, may move whole body; in waiting room, running around unfocused, not looking at toys or will not separate from parent, desperate clinging

**B. Vocalizations**

0. Can't code (child not visible or can't hear audio)

1. Reading (nonvocalizing appropriate to activity), asking questions, making comments, babbling, laughing, readily answers questions but may be generally quiet; child too young to talk in social situations or too engrossed in play to respond

2. Responding to adults but whispers, "baby talk", only head nodding

3. Quiet, no sounds or responses to adults

4. Whimpering, moaning, groaning, silently crying

5. Crying or may be screaming "no"

6. Crying, screaming loudly, sustained (audible through mask)

**C. Emotional Expressivity**

0. Can't code (can't see face or child not visible)

1. Manifestly happy, smiling, or concentrating on play

2. Neutral, no visible expression on face

3. Worried (sad) to frightened, sad, worried, or tearful eyes

4. Distressed, crying, extreme upset, may have wide eyes

**D. State of Apparent Arousal**

0. Can't Code (child not visible)

1. Alert, looks around occasionally, notices watches what anesthesiologist does with him/her (could be relaxed)

2. Withdrawn child sitting still and quiet, may be sucking on thumb or face turned into adult

3. Vigilant looking quickly all around, may startle to sounds, eyes wide, body tense

4. Panicked whimpering, may be crying or pushing others away, turns away

**E. Use of Parents**

0. Can't code (child not visible)

1. Busy playing, sitting idle, or engaged in age appropriate behavior and doesn’t need parent; may interact with parent if parent initiates the interaction

2. Reaches out to parent(approaches parent and speaks to otherwise silent parent), seeks and accepts comfort, may lean against parent

3. Looks to parents quietly, apparently watches actions, doesn't seek contact or comfort, accepts it if offered or clings to parent

4. Keeps parent at distance or may actively withdraw from parent, may push parent away or desperately clinging to parent and will not let parent go

Total adjusted score = (A/4 + B/6 + C/4 + D/4 + E/4) × 100/5
