# Supplementary material for: The Effect of Gamification through a Virtual Reality on Preoperative Anxiety in Pediatric Patients Undergoing General Anesthesia: A Prospective, Randomized, and Controlled Trial
Source: J Clin Med. 2018 Sep 17;7(9):284. doi: 10.3390/jcm7090284 (PMC6162739; doi:10.3390/jcm7090284)
Supplement: Supplementary file 1 [file jcm-07-00284-s001.zip › Supplementary Table S2.doc]

**ICC (Induction compliance checklist)**

1. Perfect induction (does not exhibit negative behaviors, fear or anxiety)
2. Crying, tears in eyes
3. Turn head away from mask
4. Verbal refusal, say “no”
5. Verbalization indicating fear or worry, “Where is mommy?” or “Will it hurt?”
6. Pushes mask away with hands, pushes nurse/anesthetist with hands/feet
7. Covers mouth/nose with hands/arms or buries face
8. Hysterical crying, may scream
9. Kicks/Flails legs/arms, arches back, and/or general struggling
10. Requires physical restraint
11. Complete passivity, either rigid or limp

Total score:
