# Supplementary material for: The Effect of Gamification through a Virtual Reality on Preoperative Anxiety in Pediatric Patients Undergoing General Anesthesia: A Prospective, Randomized, and Controlled Trial
Source: J Clin Med. 2018 Sep 17;7(9):284. doi: 10.3390/jcm7090284 (PMC6162739; doi:10.3390/jcm7090284)
Supplement: Supplementary file 1 [file jcm-07-00284-s001.zip › Supplementary Table S3.doc]

PBRS

| Cry | Tears in eyes or running down face |
| --- | --- |
| Cling | Physically holds on to parent, significant other, or nurse |
| Fear verbal | Says "I'm scared," "I'm afraid," etc. |
| Pain verbal | Says "Ow," "Ouch," "It hurts," "You're hurting me," etc. |
| Groan" | Nonverbal, vocal expresion of pain or discomfort |
| Scream | No tears, raises voice, verbal or nonverbal |
| Laugh" | Smiling with a chuckling sound |
| Stall | Verbal expression of delay ("Wait a minute," "I'm not ready yet," etc.) or behavioral delay (ignores nurse's instructions) |
| Stoic silence | Child does not respond to questions or remarks of others, may appear "trancelike" |
| Carry | Has to be physically carried into or out of room or placed on table, not because of physical inability to do so on his or her own |
| Flail | Random gross movements of arms or legs, without intention to make aggressive contact |
| Nausea verbal | Says "I'm sick," "I feel nauseous," "My stomach feels like I'm going to throw up" |
| Vomit" | Includes retching, dry heaves |
| Urinate/defecate | Soils or wets self |
| Kick | Intentional movement of leg(s) to make aggressive physical contact |
| Hit | Intentional movement of arm(s) or hand(s) to make aggressive physical contact |
| Bite | Intentional closing of jaw to make aggressive physical contact |
| Verbal hostility | Says "I hate you," "You're mean," etc. |
| Refusal position | Does not follow instructions with regard to body placement on treatment table |
| Restrain | Has to be held down because of lack of cooperativeness |
| Curse | Verbally utters profanity |
| Muscular rigidity | Any of following behaviors: clenched fists, white knuckles, gritted teeth, clenched jaw, wrinkled brow, eyes clenched shut, contracted limbs, body stiffness |
| Questions | Nondelay, information-seeking verbal behavior ("What are you doing now?" Is it over yet? etc.) |
| Emotional support | Verbal or nonverbal solicitation of hugs, physical comfort, or expression of empathy from parent,[significant other, or nurse |
| Requests termination | Verbally asks/pleads that procedure be stopped |

Total Score:
